# The Need and Opportunity to Update the Inventory of Plant Pathogenic Fungi and Oomycetes in Mexico

**DOI:** 10.3390/jof10060395

**Published:** 2024-05-31

**Authors:** Juan Manuel Tovar-Pedraza, Alma Rosa Solano-Báez, Santos Gerardo Leyva-Mir, Bertha Tlapal-Bolaños, Moisés Camacho-Tapia, Elizabeth García-León, Victoria Ayala-Escobar, Cristian Nava-Díaz, Andrés Quezada-Salinas, Víctor Santiago-Santiago, Hugo Beltrán-Peña, Maria Alondra Hernandez-Hernandez, Karla Jenifer Juárez-Cruz, Guillermo Márquez-Licona

**Affiliations:** 1 Laboratorio de Fitopatología, Centro de Investigación en Alimentación y Desarrollo, Coordinación Culiacán, Culiacán 80110, Sinaloa, Mexico; juan.tovar@ciad.mx; 2 Centro de Desarrollo de Productos Bióticos, Instituto Politécnico Nacional, Yautepec 62731, Morelos, Mexico; asolanob@ipn.mx (A.R.S.-B.); mhernandezh2309@alumno.ipn.mx (M.A.H.-H.); kjuarezc2302@alumno.ipn.mx (K.J.J.-C.); 3 Departamento de Parasitología Agrícola, Universidad Autónoma Chapingo, Texcoco 56230, Estado de México, Mexico; lsantos@correo.chapingo.mx (S.G.L.-M.); btlapal@colpos.mx (B.T.-B.); camacho.moises@colpos.mx (M.C.-T.); 4 Campo Experimental Valle del Fuerte, Instituto Nacional de Investigaciones Forestales, Agrícolas y Pecuarias, Guasave 81110, Sinaloa, Mexico; garcia.elizabeth@inifap.gob.mx; 5 Fitopatología, Colegio de Postgraduados, Campus Montecillo, Texcoco 56230, Estado de México, Mexico; ayalav@colpos.mx (V.A.-E.); cnava@colpos.mx (C.N.-D.); 6 Servicio Nacional de Sanidad, Inocuidad y Calidad Agroalimentaria, Tecámac 55740, Estado de México, Mexico; andresqs@colpos.mx; 7 Departamento de Agronomía, Instituto Tecnológico del Altiplano de Tlaxcala, San Diego Xocoyucán 90122, Tlaxcala, Mexico; santiago@colpos.mx; 8 Departamento de Ciencias Biológicas, Unidad Los Mochis, Universidad Autónoma de Occidente, Los Mochis 81223, Sinaloa, Mexico; hugobeltran@uas.edu.mx

**Keywords:** biosecurity, diversity, fungi, oomycetes, morphology, DNA sequences, collection, herbarium

## Abstract

Mexico generates specific phytosanitary regulations for each product and origin to prevent the entry of quarantine pests and/or delay their spread within the national territory, including fungi and oomycetes. Phytosanitary regulations are established based on available information on the presence or absence of these pathogens in the country; however, the compilation and precise analysis of reports is a challenging task due to many publications lacking scientific rigor in determining the presence of a taxon of phytosanitary interest in the country. This review evaluated various studies reporting the presence of plant pathogenic fungi and oomycetes in Mexico and concluded that some lists of diseases and phytopathogenic organisms lack technical-scientific basis. Thus, it highlights the need and presents an excellent opportunity to establish a National Collection of Fungal Cultures and a National Herbarium for obligate parasites, as well as to generate a National Database of Phytopathogenic Fungi and Oomycetes present in Mexico, supported by the combination of morphological, molecular, epidemiological, pathogenicity, symptom, and micrograph data. If realized, this would have a direct impact on many future applications related to various topics, including quarantines, risk analysis, biodiversity studies, and monitoring of fungicide resistance, among others.

## 1. Introduction

Crop diseases constitute a limiting factor for agricultural production, with quarantine diseases posing an exceptional threat to profitability [1,2]. Climate change and the exponential increase in global trade represent challenges for phytosanitary authorities due to the threat of new pathogens entering their territories [3]. Currently, the global trade of plant products faces significant phytosanitary challenges, among which those related to plant pathogenic fungi stand out due to the economic losses they generate, to the extent of threatening global food security [3,4,5].

Conventional methods for diagnosing plant pathogenic fungi, such as morphological identification, require prolonged time and deep taxonomic knowledge. Moreover, these techniques are often non-specific for species-level identification, making it impossible to determine intra-specific variability, which can change according to environmental conditions and crop management [6,7,8]. Since different species share morphological characteristics, identification based solely on these features is challenging and unreliable. This has led to the consolidation of the concept of “phylogenetic species” in recent years, especially phylogeny based on multigenic analyses [9]. However, establishing a universal criterion for species recognition is difficult, so the most advisable action is to generate specific criteria for fungal groups [10], which has not yet been adopted for all groups of fungi and oomycetes.

Unfortunately, phytosanitary authorities face the challenge of maintaining the plant health of the country in a context exacerbated by factors such as inefficiencies in the fungal identification system, due to the use of outdated taxonomic techniques and systems, as well as the limited information on reference biological collections and the inability to detect fungal infections of cryptic species [11]. These species are complexes composed of morphologically indistinguishable species that have been revealed through phylogenetic analysis using DNA sequence data [12,13], indicating that the actual number of species may be greater than the number of nominal species reported so far. Cryptic species have been found in various genera of plant pathogenic fungi of agricultural importance, including *Alternaria*, *Botrytis*, *Calonectria*, *Cercospora*, *Cladosporium*, *Colletotrichum*, *Erysiphe*, *Fusarium*, *Lasiodiplodia*, *Neofusicoccum*, *Neopestalotiopsis*, and *Puccinia*, among others. This underscores the need to update existing lists and databases in several countries [14,15,16,17]. Furthermore, cryptic species pose a particular problem regarding the understanding of invasion and potential quarantine procedures [11]. For example, in 1999, there were 60 species of *Phytophthora* based on morphological descriptions. By 2023, with the implementation of molecular identification, more than 200 species were recognized [18], representing a significant impact on regulators, plant trade, nurseries, and other interested parties.

Plant pathogenic fungi and oomycetes have long been documented through the concerted efforts of mycologists and plant pathologists; these records are essential for the management and prevention of plant diseases [19], as well as for compiling lists of economically or quarantine-significant pathogens. This information is integrated into databases that link the pathogen with its host range and distribution, aiming to mitigate risks associated with the importation or exportation of plant products and by-products to potential trading partners. Governments and scientists use such lists to formulate commercial quarantine policies and disease control strategies [14]. Additionally, it is crucial that inventories and databases of plant pathogens are supported by herbarium specimens, living cultures, and DNA sequence databases [20]. However, it is often challenging to extract DNA from herbarium specimens for the validation of identifications [21,22], and since morphology alone does not differentiate taxa within species complexes, researchers need to recollect diseased specimens, perform isolations, and molecularly identify them to validate specimen identities. This need is further justified as maintaining living cultures in plant pathology is not a common practice [16,20,23] due to the high time, space, and financial resource consumption involved.

Currently, there are international standards and technologies that provide appropriate information to establish biosafety and quarantine protocols (https://www.cdfa.ca.gov/plant/regs_pestrating.html, accessed on 25 May 2024); however, the scientific community and regulatory entities in general do not work in a coordinated manner, limiting the exchange of information expediently. Therefore, it is urgent to establish and adopt harmonized international agreements to modernize phytosanitary systems focused on preventing risks related to the dissemination of plant pathogens associated with trade, in addition to reconsidering the way fungal pathogens are identified and detected [11].

The timely identification of new and re-emerging plant pathogens depends on rapid access to current diagnostic protocols, which integrate different techniques for identification [24]. Additionally, access to databases and collections representing the known diversity of pathogens, along with the genotypic, phenotypic, and epidemiological data associated with them, is crucial. Therefore, registering such data in a format that can be easily accessible for quick assessment of potential risk is essential [25].

Plant pathogen culture collections, serving as libraries of the genotypic and phenotypic diversity of previously studied pathogens, constitute invaluable resources for advancing future research in plant pathology [26,27]. Enhancing our capability for pathogen detection and disease diagnosis will significantly increase the likelihood of containing and eradicating high-risk pathogens [28,29,30,31]. However, several of these pathogen culture collections are relatively small and often focus on a particular genus of pathogen [25]. Furthermore, such biological resource centers typically originated as personal collections and are housed within academic institutions where they are usually maintained without a permanent funding source [11,25].

Regarding genome sequencing, current studies have focused on specific isolates of a particular species due to the pathogen’s importance and the high costs associated with these technologies. However, it is expected that genome sequencing costs for fungi will decrease considerably in the short term, allowing the technique to be routinely implemented in more laboratories [32,33]. However, complete knowledge of genetic composition is insufficient to understand the pathogenic potential within a species because crop losses are caused by populations of isolates that vary in virulence, host range, compatibility type, chemical resistance, and/or toxin production [25]. Therefore, obtaining DNA sequences from fungal species found in culture collections that represent certain phenotypic, spatial, and temporal diversity is essential [23,25].

Often, nomenclature is confused with taxonomy; however, it is necessary to recognize that they are different activities. Nomenclature refers to the application of names to biological units (taxa), while taxonomy determines the validity of formal scientific names [15]. In recent years, several changes have occurred in the taxonomy and nomenclature of plant pathogenic fungi and oomycetes [34], making it crucial to incorporate these modifications into databases to develop precise quarantine or regulatory strategies. Some of these changes in taxonomy and nomenclature have been extensively discussed at various specialized mycological events and in several publications [35,36,37,38]. In this regard, the GOPHY database (http://www.plantpathogen.org, accessed on 25 May 2024) was established to provide updated descriptions of each genus of plant pathogenic fungi, ensuring the application of the genus name whenever possible. It also provides a general description of currently accepted species, their DNA barcodes, and relevant literature [17,39,40,41].

On the other hand, regulatory policies concerning plant pathogens traditionally rely on names; however, in many cases, these names have not been updated [34], leading to inaccurate representation of information associated with a particular pathogen. This is because there are excessively long lists of pathogen names, including synonyms, which persist in regulated or quarantine pathogen lists at the national level. However, these lists are often only found in inaccessible national databases or government publications, and commonly, they are not linked to relevant data or consistent with modern taxonomic concepts [11].

**Figure 1:**
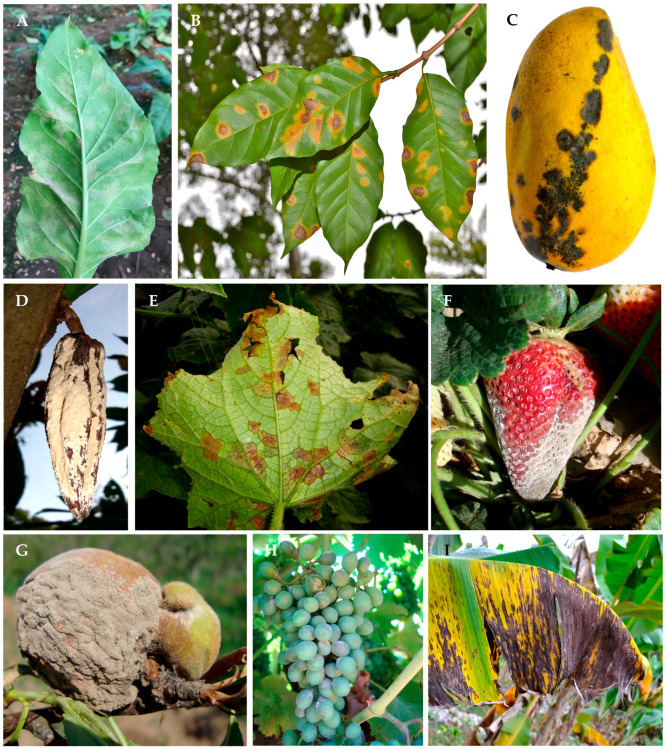
Signs and symptoms caused by fungi and oomycetes in agricultural crops of importance to Mexico. (**A**) Tobacco blue mold caused by *Peronospora tabacina*. (**B**) Coffee rust caused by *Hemileia vastatrix*. (**C**) Mango anthracnose caused by *Colletotrichum siamense*. (**D**) Cacao moniliasis caused by *Moniliophthora roreri*. (**E**) Cucumber downy mildew caused by *Pseudoperonospora cubensis*. (**F**) Strawberry gray mold caused by *Botrytis cinerea*. (**G**) Brown rot caused by *Monilinia fructicola*. (**H**) Powdery mildew on grapes caused by *Erysiphe necator*. (**I**) Black Sigatoka of banana caused by *Pseudocercospora fijiensis*.

**Figure 2:**
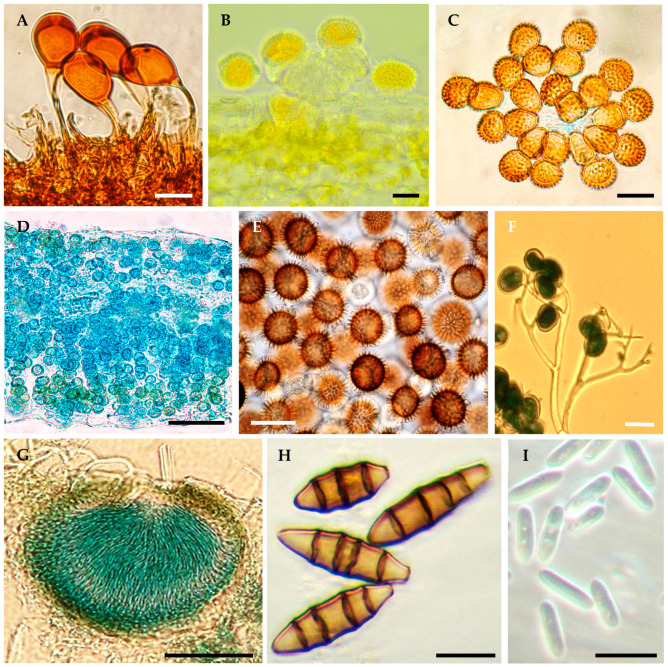
Reproductive structures of some fungi and oomycetes causing diseases in plants of agricultural importance in Mexico. (**A**) Teliospores of *Uromyces viciae-fabae*. (**B**) Urediniospores of *Hemileia vastatrix*. (**C**) Teliospores of *Tranzchelia discolor*. (**D**) Teliospores of *Entyloma australe*. (**E**) Teliospores of *Tilletia indica*. (**F**) Sporangiophores and sporangia of *Peronospora sparsa*. (**G**) Pycnidium of *Zymoseptoria tritici*. (**H**) Conidia of *Wilsonomyces carpophilus*. (**I**) Conidia of *Colletotrichum siamense*. Bars = 20 µm (**A**–**C**,**F**,**H**,**I**), 50 µm (**D**,**E**,**G**).

The aim of this review is to analyze the status of the inventory of fungal and oomycete plant pathogenic in Mexico. Additionally, some examples of lists of fungi and oomycetes causing plant diseases in Mexico that lack scientific support are presented, highlighting the need and opportunity to update the inventory of these pathogens. This can be achieved through the establishment of a National Collection of Fungal Cultures and a National Herbarium for obligate parasites, as well as the creation of a National Database of Fungal and Oomycete Phytopathogens. This database should be supported by a combination of morphological, molecular, epidemiological, pathogenicity, symptomatology (Figure 1), and micrograph data (Figure 2).

## 2. Inventory of Plant Pathogenic Fungi and Oomycetes in Mexico

In Mexico, there are reports of fungal and oomycete pathogens causing diseases in cultivated plants. These reports can be classified into three categories:

(a) **Reports of diseases and phytopathogens based on research that includes morphological characterization, DNA sequence analysis, and verification of pathogenicity through artificial inoculations, except for some obligate parasites**. Generally, these studies report the presence of a fungal or oomycete phytopathogen based on the international norms established in ISPM8 [42], so they should be considered valid reports of the presence of the pathogen in a new territory.
(b) **Lists of diseases and plant pathogens based on literature reviews of studies published in scientific journals and books in the country**. León-Gallegos (1978) [43], in his book titled “Enfermedades de cultivos en el Estado de Sinaloa”, conducted a literature review and compiled information regarding the symptoms and control of the main diseases caused by fungi, oomycetes, viruses, and bacteria in various economically important crops. However, it cannot be considered a conclusive report on the presence of such species of plant pathogens, as it lacks the elements described in the previous section. Another example is found in the book “Hongos Fitopatógenos” published by Romero-Cova (1988) [44], which describes the main morphological characteristics used for the identification of various fungi and oomycetes affecting major economically important crops. The work also mentions their distribution, host range, life cycle, and control. However, much of the taxonomic information presented in this work is based solely on a compilation of keys and descriptions from specialized books or journals, so the information provided by this work also does not comply with ISPM 8, and the reports should not be considered valid. However, it is worth noting that the book provides detailed morphological identification of various specimens supported by excellent photomicrographs. More recently, Fernández-Pavía et al. (2015) [45] published a book titled “Enfermedades de Especies Vegetales en México”, which presents an excellent compilation of publications where the presence of plant pathogens in Mexico was recorded or mentioned. However, in this book, some lists and catalogs of fungal and oomycete pathogens were considered, which were not identified through morphological characterization and whose pathogenicity was not verified. Therefore, this source of information should be considered with some reservations.
(c) **Lists or catalogs of diseases and plant pathogens without scientific support**. These are reports that were not verified by morphology, nor confirmed through DNA sequence analysis, and certainly not subjected to pathogenicity tests. One example is the “Primer catálogo de enfermedades de plantas mexicanas” published by García-Alvarez (1976) [46], which is one of the most extensive lists of plant diseases in Mexico; however, this catalog was generated without scientific evidence or support. Ayala-Escobar et al. (2013), Solano-Báez et al. (2017), Márquez-Licona et al. (2018), and Nieto-López et al. (2018) [47,48,49,50] mentioned that the catalog published by García-Alvarez (1976) [46] lacks morphological characterization data of the causal agents mentioned in the list. Consequently, this list does not comply with ISPM 8 [42], and should not be considered an official report of plant pathogenic species in Mexico.

Based on this review, it is proposed to completely exclude the list published by García-Alvarez (1976) [46] from the inventory of fungal and oomycete pathogens in Mexico, based on the following grounds: lack of precise identification using morphological tools, as well as the absence of pathogenicity tests. For this reason, it is of utmost importance to consider only as valid reports of any plant pathogen in Mexico those studies of fungal and oomycete identification classified in the first category proposed in this work, i.e., only those reports based on studies where pathogenicity was evaluated, and specimens were identified through morphological and molecular data analysis.

Finally, it is worth noting that various reports or records of fungal and oomycete in Mexico have been published in abstract format and in proceedings of scientific congresses. However, these reports should not be considered conclusive and should only be accepted until they are published in the form of a note, short communication, or scientific article in a peer-reviewed specialized scientific journal. Likewise, there are some publications in which the results of other studies were misquoted, which can have negative repercussions on commercial treaties of importance to Mexico. Therefore, it is essential to resort to the original sources, determine their relevance according to the criteria, and subsequently include or exclude a certain pathogen in the lists of pests for a region.

## 3. Plant Pathogen Fungal Collections and Plant Pathology Herbaria

Biological collections are essential components of research conducted in biodiversity research centers [51,52,53], particularly in Systematics, as they house representative specimens of organisms, populations, and species collected at specific times and locations. Each accession in biological collections represents a historical record of the biodiversity of a particular region or country, thus expanding research possibilities in ecology and evolutionary biology, among others [54]. Therefore, each accession should be associated with additional metadata such as photographs, illustrations, and bibliography, among others [55].

Mexico is a country with a vast diversity of organisms [56]. It ranks fifth globally in terms of diversity due to its number of endemic species and accounts for 10% of the world’s terrestrial diversity [57]. The geography, topography, and variety of climates in Mexico have favored environmental conditions that promote diverse habitats [58]. In Mexico, scientific collections are primarily concentrated in higher education institutions, research centers, and government agencies, with their main objectives being research, teaching, outreach, and serving as reference collections [55]. However, many others are private collections belonging to researchers, which are not internationally registered but undoubtedly represent an important part of Mexican biodiversity. In some cases, these collections house unique specimens that are not found in any other collection. Regarding fungi, data estimate that Mexico is home to at least 200,000 species of fungi, of which only 5% are known [59]. In this regard, Guzmán (1994) [60] conducted an analysis of dry and living collections in Mexico and emphasized that they are small and poorly representative compared to the vast mycological diversity in the country. He also mentioned that many Mexican fungal collections are in poor condition, and the specimens lack the necessary data for accurate identification.

More recently, Koleff et al. (2016) [61] published a list of biological collections from institutions located in different states and registered with the National Commission for the Knowledge and Use of Biodiversity (CONABIO) (www.biodiversidad.gob.mx, accessed on 25 May 2024). The list shows that there are 27 collections of microorganisms, 15 collections or fungal repositories, 2 fungal herbaria, 1 plant pathology herbarium (CMPH), and 1 collection of phytopathogenic fungi (IFIT); the latter two belong to the Institute of Phytosanitary, Campus Montecillo of the Colegio de Postgraduados. According to CONABIO (2012) [62], the IFIT collection of plant pathogenic fungi comprises 3300 specimens, making it the most extensive collection of plant pathogenic fungi in Mexico. However, it lacks a database containing confirmed identifications through DNA sequence analysis, as well as images of disease symptoms or photomicrographs of fungal structures. The Servicio Nacional de Sanidad, Inocuidad y Calidad Agroalimentaria (SENASICA) has an entomological collection (insects and mites) and a collection of strains of plant pathogenic bacteria belonging to the Dirección General de Sanidad Vegetal [61,62]. Additionally, it has a reference collection of fungi and oomycetes; however, this collection is not officially registered and does not have a specialized herbarium for diseased plants.

In general, there is a lack of a national collection of cultures and herbarium specimens meticulously characterized by morphology and sequencing according to international standards. These reference specimens and cultures are essential for establishing reliable and accurate identification systems, and their loss would pose a significant hindrance to future generations of mycologists, plant pathologists, and other end-users [11]. It is worth noting that some countries with extensive collections specialized in plant pathogenic fungi and oomycetes include Germany, Australia, Canada, the United States, France, India, Japan, the Netherlands, the United Kingdom, and South Africa.

It is worth mentioning that there are various methods to preserve cultures of fungi both in the short term and in the long term. Some of the most used short-term methods include subculturing, storage in mineral oil, and deep freezing. Meanwhile, the main long-term preservation methods are lyophilization and cryopreservation. Other methods also used include culturing in sterile distilled water, silica gel, sand, desiccation on filter paper, and vitrification of obligate parasites and fruiting bodies of mushroom fungi [26,63,64]. However, no single method can be successfully applied to all fungi, so those responsible for culture collections must select the most appropriate method for each fungus and oomycete. In many cases, it is recommended to preserve a specimen using different methods to ensure preservation [26].

Regarding herbarium specimens, these are used as reference material for routine identification of plant diseases, for research on the classification of new plant pathogenic fungi, as well as for providing lists of plant diseases and information related to quarantine or regulation aspects. Some of the most outstanding herbaria specialized in plant pathology are in Germany, Australia, the United States, Japan, the United Kingdom, and the Netherlands.

Plant pathogen databases are of great importance due to the extensive information they contain, which can be accessed quickly and easily without the need to examine the physical specimen. Additionally, they are useful for generating distribution maps of specific plant pathogens, as well as lists of pathogens present on specific hosts. Moreover, they contain images of disease symptoms, photomicrographs of fungal structures, and DNA sequences of reference pathogen species. Some successfully generated phytopathogen databases are in Australia, the United States, Japan, and New Zealand. These databases are extremely important tools in the categorization of plant pathogens and the identification of potential phytosanitary measures, thus having a significant impact on global and commercial biosecurity [14,20,23]. Among these, the Rust Fungi of Australia (collections.daff.qld.gov.au/web/key/rustfungi) and Smut Fungi of Australia (collections.daff.qld.gov.au/web/key/smutfungi) databases stand out, containing detailed distribution data, taxonomic keys, new detections and taxa, and images of symptoms and fungal structures observed under light and/or scanning microscopy [65].

It is important to note that there are few morphological characters that help discriminate between species within some genera of fungi and oomycetes, making it difficult to distinguish some species based on morphology alone. Additionally, some morphological characters of pathogens vary depending on the host, environmental conditions, and culture conditions, which complicates their accurate identification. This suggests that DNA sequences provide a suitable and effective means to distinguish species and populations of fungi, regardless of their morphological plasticity, ability to be cultured, or their pathogen–host interactions [20,23,25,66]. However, this does not imply that molecular markers will replace classical phenotypic methods, as genotypic data without biological context have limited value. Therefore, both approaches should be considered complementary tools for the diagnosis and evolutionary studies of plant pathogens [23,25].

The analysis of the internal transcribed spacer (ITS) region of ribosomal DNA has been widely used to distinguish or separate between fungal species. However, it has been observed that the ITS region does not always provide good resolution at the species level, although it will almost always provide sufficient resolution to support species assignment at the species complex level [23,66]. Therefore, nowadays, more genes or regions are being used (Table 1), often including a mix of mitochondrial, nuclear, ribosomal, and protein-coding genes, which are analyzed using different approaches or criteria such as Maximum Parsimony, Maximum Likelihood, and/or Bayesian Inference in order to determine the phylogenetic relationships of some of the major genera of plant pathogenic fungi [15,23]. For example, in Australia, the Biosecurity Bank (www.biosecuritybank.com, accessed on 25 May 2024) provides a reference DNA collection of a wide range of agricultural plant pests and pathogens for molecular analysis and taxonomic verification. This is achieved solely through the combination of molecular and morphological data, allowing plant pathogens within Australia’s borders to be reliably identified [20].

## 4. Reports of Plant Pathogens in Mexico and Their Implications in International Databases

The International Plant Protection Convention (IPPC) (https://www.ippc.int/es/, accessed on 25 May 2024) is an intergovernmental treaty aimed at protecting plant health globally. It is responsible for developing, adopting, and promoting the implementation of International Standards for Phytosanitary Measures (ISPMs) as a key strategy to safeguard food security, facilitate safe trade, and protect the environment on a global scale. Regional plant health organizations, such as NAPPO (North American Plant Protection Organization) and OIRSA (Organismo Internacional Regional de Sanidad Agropecuaria), operate under the umbrella of the IPPC. These regional organizations engage national plant protection agencies, such as SENASICA in Mexico and USDA APHIS in the United States, to monitor and ensure plant health within their respective countries.

Regional plant health organizations have among their main objectives the establishment of cooperation links regarding plant protection, either regionally or internationally. Among these organizations, the European and Mediterranean Plant Protection Organization (EPPO) stands out. It is an international organization responsible for the cooperation and harmonization of plant protection in the European and Mediterranean regions. Additionally, CABI, which stands for Centre for Agricultural Bioscience International, is an intergovernmental organization that conducts scientific research worldwide on agricultural and environmental issues. Its specific projects include pest diagnosis, development of control methods, and teaching farmers best practices in their field.

Another example is the North American Plant Protection Organization (NAPPO), whose main objective is to promote and facilitate cooperative activities among its member countries (Canada, the United States, and Mexico) to prevent the entry, establishment, and spread of regulated pests in the NAPPO region and to limit the economic impacts of pests. In addition to establishing guidelines, agreements, standards, treaties, and research development in plant health, these organizations provide documentation and information services on plant protection, including records of the presence or absence of pests with an emphasis on those of regulatory interest. These pest records can be presented in the form of lists of regulated pests, monographs, and databases.

The importance of the resources lies in their status as scientifically validated sources of information, as they are mostly based on scientific articles, books, and official reports issued by different countries. They also represent a robust source of information that facilitates the establishment of phytosanitary requirements for importation and helps identify potential threats from invasive species to a country, state, or province. These databases are used by risk assessors, phytosanitary protection officials, quarantine officers, protected area managers, and researchers; they can consult literature on the spread, distribution, detection, and control of pests, as well as identification keys, image libraries, and phytosanitary information.

Therefore, the importance of reports on plant pathogens in Mexico and their implications in international databases lies in the fact that this is the available information for countries when conducting Pest Risk Analyses that may be associated with plant products and by-products from Mexico [75]. Such information is considered valid from various sources (official reports, literature reports, as well as lists or catalogs of diseases and pathogens), the scientific rigor applied in their elaboration and integration of which is often unknown in detail; that is, the scientific validity of the information and its sources is not regularly analyzed and evaluated in the Pest Risk Analysis studies conducted by other countries on Mexican products. This information, even if lacking scientific support, can be highly valued for establishing strict, restrictive, or difficult-to-comply-with regulatory measures for vegetable products and by-products [76,77]; this has implications for exportation, or in the worst case, the closure of markets for plant products. This should raise awareness to update pathogen reports based on studies of morphological characterization, DNA sequence analysis, and pathogenicity testing under scientific scrutiny.

## 5. Conclusions

This review highlighted the urgent need and opportunity to establish a National Fungal Culture Collection and a National Herbarium for obligate parasites, linked to a National Database of Plant Pathogenic Fungi and Oomycetes in Mexico, supported through the combination of morphological, molecular, epidemiological, pathogenicity, symptom, and micrograph data. Such a collection will be of crucial importance, as it will allow for the maintenance of viable and pure cultures of fungi and oomycetes to conduct various studies that will facilitate the management of plant diseases caused by these pathogens. Some examples of the expected applications and uses of the proposed Collection, Herbarium, and Database are listed below:

(a) Distribution of agricultural important fungi and oomycetes in Mexico.
(b) Detailed information (common name, species, and variety or cultivar of the host plant; scientific name of the pathogen; geographic coordinates, date of collection, collector’s name, identifier’s name, and photographs of symptoms in the field; microphotographs of characteristic structures of the pathogenic fungus, etc.)
(c) Assessment and recording of damage and impacts, by regions or climatic conditions.
(d) Prevalence of the pest in the regional context, and epidemiological studies.
(e) Ease of exchanging specimens of fungi and oomycetes with herbaria or plant pathogens collections from other countries.
(f) Monitoring of fungicide resistance in reference populations of fungi.
(g) Modification or adaptation of biosecurity measures in Mexico.
(h) Evaluation of tolerance or resistance of varieties or cultivars of plant species.
(i) Population structure of fungal and oomycete species.
(j) Studies on comparative genomics, transcriptomics, proteomics, and metabolomics.

## 6. Future Directions

A significant portion of the needs outlined in this review are addressed by the establishment of a Specialized Center for Mycological Research dedicated to conducting research on the exploration, study, preservation, bioprospecting, and utilization of fungal diversity, aimed at generating solutions to the current challenges faced by our society. The initial perspective regarding the establishment of this center suggested it be a publicly funded research institution, directly financed by the Federal Government. However, given the acknowledged administrative challenges and historical budgetary constraints on research, it is considered more advantageous for the government to provide only the seed capital. Consequently, the center is proposed to operate as a private research institution.

## Figures and Tables

**Table 1:** Recommended molecular markers for genus- and species-level identification of some major plant pathogenic fungi and oomycetes.

Pathogen	Marker for Genus-Level Identification	Marker for Species-Level Identification	Reference
*Alternaria*	LSU, SSU	ITS, GAPDH, RPB2, TEF1	[67]
*Bipolaris*	LSU	ITS, GAPDH, TEF1	[17,66,67]
*Botrytis*	ITS	GAPDH, RPB2, HSP60, NEP1, NEP2	[66,68,69]
*Cladosporium*	LSU	ACT, TEF1, TUB2	[17]
*Colletotrichum*	ITS	ACT, APMAT, APN2, CAL, CHS-1, GAPDH, GS, HIS3, SOD2, TUB2	[17,66,70]
*Curvularia*	LSU, ITS	ITS, GAPDH, TEF	[17,66,67]
*Diaporthe*	ITS	CAL, HIS3, TEF1, TUB2	[41,66]
*Elsinoe*	ITS	RPB2, TEF1	[67]
*Erysiphacea*	ITS	ITS, LSU	[71,72]
*Entyloma*	ITS	ITS	[67]
*Fusarium*	ITS	TEF1, RPB1, RPB2, TUB2, cmdA	[73] http://isolate.fusariumdb.org/ (accessed on 25 May 2024)
*Gaeumannomyces*	LSU	ITS, TEF1, RPB1	[41]
*Lasiodiplodia*	ITS	TEF1, TUB2	[66,74]
*Monilinia*	ITS	TEF1	[17]
*Neofusicoccum*	SSU, LSU	ITS, TEF1, TUB2, RPB2	[17,66,67,74]
*Phyllosticta*	LSU	ITS, ACT, GAPDH, TEF1	[41,66,67]
*Phytophthora*	ITS	LSU, TUB2, COX2, NAD9, RPS10	[66] www.phytophthoradb.org (accessed on 25 May 2024)
*Puccinia*	ITS	ITS, LSU	[17,66]
*Pyricularia*	LSU	ITS, ACT, CAL, RPB1	[41]
*Pyrenophora*	LSU	ITS, GAPDH	[66]
*Pythium*	LSU, SSU	ITS, COX1, COX2	[66]
*Tilletia*	LSU	ITS	[67]
*Ustilago*	LSU	ITS	[66]
*Venturia*	LSU, SSU	ITS, TEF1, TUB2	[17,67]
*Verticillium*	ITS	ACT	[66]
*Wilsonomyces*	LSU	ITS, TEF	[17]

LSU = 28S large subunit of the nrRNA gene; SSU = l8S small subunit of the nrRNA gene; ITS = internal transcribed spacer regions 1 & 2 including 5.8S nrRNA gene; TEF1 = partial translation elongation factor 1-α; BT2 = β-tubulin 2; GAPDH = glyceraldehyde-3-phosphate dehydrogenase; RPB1 = partial RNA polymerase II largest subunit; RPB2 = RNA polymerase II 2nd largest subunit; HSP60 = Heat-shock Protein 60; NEP1 = Necrosis and ethylene-inducing peptide 1; NEP2 = Necrosis and ethylene-inducing peptide 2; ACT= Actin; ApMat = Apn2-Mat1-2 intergenic spacer and partial mating type (Mat1-2) gene; APN2 = DNA-(apurinic or apyrimidinic site) lyase APN2; CAL and cmdA = Calmondulin; CHS-1 = chitin synthase; GS = Glutamine synthetase; HIS3 = Histone; SOD2 = manganese-superoxide dismutase; COX1 = cytochrome c oxidase subunit I; COX2 = cytochrome c oxidase subunit 2; NAD9 = mitochondrial gen nad9; RPS10 = ribosomal protein S10.

## Data Availability

Not applicable.
